# On Peripheral Neuritis

**Published:** 1893-09

**Authors:** 


*On Peripheral Neuritis.* By JAMES ROSS, M.D., AND JUDSON S. BURY, M.D. With illustrations. Pp. 424. London: CHARLES GRIFFIN & COMPANY, LIMITED. 1893.

The larger part of this work is from the pen of the late Dr. Ross, and we here gain additional evidence of the great loss the medical profession has to deplore in the death of so accurate and scientific an observer. To our mind Dr. Ross latterly occupied a unique position amongst neurologists. He united a wide knowledge of the older writers, and of their methods of work, to an equally extensive and thorough acquaintance with the great advances made during the last thirty years in the theory and practice of nervous diseases. To his ability to bring to bear on recent discoveries all that was true and worthy to survive from the past, we think he owed that far-reaching power of generalisation and the broad grasp of his subject which distinguish his writings. For his was one of those rare minds that can give due weight to minutiæ of a subject—and in neurology much depends on the duly weighing and balancing of points of detail—without thereby losing sight of its general relations. His writings are marked by the presence of that culture which is so often wanting in scientific works to-day. All these qualities are exemplified—as witness the very interesting criticism on the character of Macbeth—in the present volume, which more immediately concerns us here.

First of all, this book is a record of much original work, and at the same time it gives a complete account of all that has so far been done in the matter of peripheral neuritis. With this treatise the first stage in the investigation of these diseases is brought to an end. To a large extent the anatomy and the etiology of peripheral neuritis are now known; the next stage will be the placing of the disease in its proper relations to other nervous disorders. Dr. Ross first considers Landry's paralysis, and gives an analysis of ninety-three published cases. He shows on how slender a foundation of actual pathological observation the current theory, which places the seat of the disease in the anterior cornua of the spinal cord, rests. It is also remarkable to find what little satisfactory evidence there is for the general text-book statement that the electrical reactions are unaltered. Dr. Ross contends that the majority of cases of Landry's paralysis are due to acute peripheral or multiple neuritis; he shows that the pathological observations at our disposal are insufficient and incomplete, but that so far as they go, the spinal cord has not been found to be implicated, whilst in nine cases in which the nerves were properly examined there was neuritis in six; he points out that in the very acute cases we are not likely to find changes in any part of the nervous system which can be recognised by the present methods of research, but that in cases which run a longer course there is neuritis, while finally there is an identity of the clinical symptoms and course of the disease in Landry's paralysis and in undoubted cases of peripheral neuritis from diphtheria, alcohol, &c. We may allow the cogency of these arguments, and yet submit that they do not cover the whole ground, and that in some cases the morbid process does take place in the cord itself.

Very valuable sections on subacute and chronic neuritis follow, and we may say here that under neuritis the authors include any process, inflammatory or degenerative, in which the peripheral nerves are damaged. We would specially call attention to the descriptions of the contractions and other deformities produced in the course of multiple neuritis which are contained in these sections. Then follows a masterly account of alcoholic neuritis, of which the author had a very large experience, and we may perhaps here select as particularly good the section on the psychical disorders met with in these cases. A great deal of the next sections on the effects of other diffusible stimulants in producing neuritis covers new ground, and with the following chapter on diphtheritic paralysis Dr. Ross's own work comes to an end.

If the book ended here it would be manifestly incomplete, as there would be no account of the many other important conditions under which neuritis occurs. The difficult task of rendering this study of neuritis a complete one—of finishing another man's work, or rather contributing one-third part to it—has been undertaken with great ability and signal success by Dr. Judson Bury. He has added the account of the peripheral neuritis caused by the acute specific fevers; by syphilis, tubercle, and leprosy; by the metallic poisons, and by the poisons arising in the disordered blood states; and to him is also due a suggestive and thoughtful chapter on the general pathology of peripheral neuritis. We think that the neuritis which has been shown by the labours of Leyden, Déjerine, and others to form so important a part of the symptom-complex of many cases of tabes dorsalis deserves to be treated more fully in future editions than it is in this present one. Perhaps also the latter part of the book is, in proportion to the other matter in the text, a little overladen with accounts of individual cases.

These are minor faults, and taking the book as a whole, we have nothing but praise for the admirable manner in which it has been compiled, and have no hesitation in saying that it is the best work on the subject. We have in it a complete account of the present state of knowledge on peripheral neuritis, and also an indication of the lines on which further advance is to be made. The full bibliography to each section is a feature of the work which much enhances its value, and makes the book a satisfactory work of reference. The type is clear and good, and the general get-up of the book excellent, except that pp. 257—272 seem to have become hopelessly mixed in binding.

